# Tension Estimation in Anchor Rods Using Multimodal Ultrasonic Guided Waves

**DOI:** 10.3390/s25061665

**Published:** 2025-03-07

**Authors:** Thilakson Raveendran, Frédéric Taillade

**Affiliations:** Performance, Risque Industriel et Surveillance pour la Maintenance et l’Exploitation (PRISME) Department, R&D Division, EDF, 78400 Chatou, France; frederic.taillade@edf.fr

**Keywords:** anchor rods, guided waves, tension, post-tensioned, dispersion curves, time–frequency analysis

## Abstract

The diagnosis of post-stressed anchor rods is essential for maintaining the service and ensuring the safety of *Electricité de France* (EDF) structures. These rods are critical for the mechanical strength of structures and electromechanical components. Currently, the standard method for estimating the effective tension of post-stressed tie rods with a free length involves measuring the residual force using a hydraulic jack. However, this method can be costly, impact the structure’s operation, and pose risks to employees. Until now, there has been no reliable on-field approach to estimating residual tension using a lightweight setup. This research introduces a nondestructive method using multimodal ultrasonic guided waves to evaluate the residual tension of anchor rods with a few centimeters free at one end. The methodology was developed through both laboratory experiments and simulations. This new method allows for the extraction of dispersion curves for the first three modes, bending, torsional, and longitudinal, using time–frequency analysis and enables the estimation of the steel bar’s properties. Future work will focus on applying this methodology in the field.

## 1. Introduction

The diagnosis of passive and prestressed anchor rods is a critical issue for the operation and safety of *Electricité de France* (EDF) structures [1]. Anchor rods play a key role in ensuring the mechanical stability of structures and electromechanical components.

An anchor rod is typically composed of a metal bar, an assembly of wires forming a strand, or twisted wires. Depending on the configuration, the rod may be accessible from one side or both sides or be fully embedded in the structure. The length can be up to several tens of meters.

With aging, the performance of these old anchors declines, increasing the probability of accidents due to tension loss. Inspection methods are, therefore, essential to establishing an optimized maintenance program and ensuring the safety of these structures. Currently, to estimate the effective tension in prestressed rods with a free length, the reference method involves pulling on the rod using a hydraulic ram to directly assess its tension [2]. However, implementing this method can be costly, impact the structure’s operation, and expose workers to risks. Moreover, this approach is not applicable to fully grouted anchors.

A lightweight and non-destructive inspection methodology is preferred to assess the integrity and conservation state of the reinforcement and, in the case of prestressed anchor rods, to estimate the effective tension. This type of method is preferable for increasing the frequency of inspections and, thus, improving structural monitoring.

In the literature, there are numerous studies on the effect of corrosion, which leads to thickness loss [3]. For instance, guided waves are used to inspect reinforcements embedded in concrete to detect this corrosion effect [4,5]. Various studies show that there are modes sensitive to corrosion and that they must be chosen wisely according to the nature of the defects sought. An indicator related to the amplitude of the signals or the time of flight for monitoring corrosion has been proposed [6,7]. However, that analysis often relies on transmission measurements with access on both sides. Furthermore, in laboratory conditions, the corrosion is generated in an accelerated and controlled manner using a permanent device [8].

Studies have also been carried out to inspect the health state of tensioned cables [9,10], which take the form of twisted wires constituting a strand. An initial experimental validation was carried out under laboratory conditions [11]. The simulated dispersion curves corresponding to the longitudinal modes were converted into time relative to the distance traveled by the wave, and the received signal was processed using a time–frequency analysis, allowing for a comparison of the simulated and measured results. A good agreement between the experimental and simulation results was obtained. Note that the transmitter was positioned at about one-quarter of the bar length, and the receiver was placed in the middle, which is not possible in our case, where often, only the end of the bar is accessible.

Regarding the estimation of tie rod tension, a related issue found in the literature is the measurement of stress in bolted assemblies with ultrasonic methods [12,13]. The residual tension is estimated by taking advantage of the acoustoelastic effect [14]: as the tension in the bar increases, the wave propagation speed changes compared to the unstressed state. Numerous studies have demonstrated the relationship between time-of-flight measurements and tension [15,16]. However, the acoustoelastic effect influences the third-order elastic constants [17], resulting in only a minor variation in the velocity of ultrasonic waves due to the applied stresses.

In theory, it is possible to estimate tension based on time-of-flight measurements. However, a high accuracy is required when measuring the time-of-flight of waves. Moreover, the time of flight depends on other factors, particularly the material properties and the length, which are often unknown. Therefore, in practice, a calibration step is required before using this method.

Similarly, in guided structures, certain modes can be identified as sensitive to tension and could be used to measure the acoustoelastic effect and estimate tension [18,19]. The sensitivity of ultrasonic waves to tension is better when the wave polarization is in the same direction as the loading axis [15].

Y. Wu et al. [20] notably described the influence of tension on the theoretical dispersion curves associated with the modes of a bar. Measurements were then carried out in transmission for different frequencies for the first two longitudinal modes. Good coherence between the measurements and simulations was observed. However, the results were obtained by estimating arrival times from correlations between measurements with and without tension, and the bar length was known. This study underlines that, given the difference between the theoretical model and the actual material properties, the technique in its current state cannot be directly applied in situ and suggests a calibration step.

These studies confirmed the sensitivity of guided waves to the sought tension. However, an absolute measurement of tension using ultrasonic waves has never been achieved to the best of our knowledge. The major issue lies in a lack of knowledge of steel properties. To tackle these difficulties, the solution implemented in the literature consists of performing a calibration step without tension.

However, studies generally exploit only one operating point, i.e., on a given mode at a given frequency. One possibility would be to develop a methodology that exploits the multimodal nature of guided waves over a wide range of frequencies.

A classic method to access experimental dispersion curves is to create a measurement line and calculate the 2D Fourier transform or an equivalent [21,22]. Numerous ultrasound studies have used this method with a wide range of application domains, from the medical field—for example, monitoring blood pressure [23,24]—to non-destructive testing in civil engineering [25,26]. However, implementing these methods on tie rods is complicated since only the end of the bar is accessible, at best, for a few centimeters. Some articles propose extracting dispersion curves experimentally from a limited number of measured signals [27,28]. Here, we propose implementing a time–frequency analysis with measurements only at the end of the bar. The challenge is to successfully excite and measure different modes of interest accurately enough to determine the steel properties and effective tension.

In this study, we propose using low-frequency modes, on the order of a few tens of kHz, to overcome difficulties related to attenuation. The aim of this study is to first demonstrate, in controlled laboratory conditions, the feasibility of this approach based on the reconstruction of dispersion curves associated with different modes with access only to the end of the bar. The successful reconstruction of dispersion curves under laboratory conditions would enable us to identify the unknown properties of the tie rod and estimate the effective tension.

## 2. Materials and Methods

### 2.1. Dispersion Curves Simulations

The propagation of waves in a rod is described by dispersion curves [29]. The propagation speed depends on the mode considered and the frequency. These dispersion curves are influenced by the material properties and the diameter of the rod. In this study, the dispersion curves are computed using the SAFE method [30] implemented in COMSOL Multiphycis 6.1^®^.

When axial stress along the waveguide axis (z-axis) is considered with isotropic properties in the x–y plane, the stiffness matrix is written as

(1) $$K=\left[\begin{array}{cccccc} C_{22} & C_{23} & C_{12} & 0 & 0 & 0\\ C_{23} & C_{22} & C_{12} & 0 & 0 & 0\\ C_{12} & C_{12} & C_{11} & 0 & 0 & 0\\ 0 & 0 & 0 & C_{55} & 0 & 0\\ 0 & 0 & 0 & 0 & C_{55} & 0\\ 0 & 0 & 0 & 0 & 0 & \frac{1}{2}\left(C_{22}-C_{23}\right) \end{array}\right].$$

To introduce the effect of stress, σ, in the rod on the dispersion curves, the stiffness matrix coefficients are modified by incorporating Murnaghan coefficients, l, m, n (from the field of acousto-elasticity).

The expressions for these coefficients are provided by the following equations [31]:

(2) $$C_{11}=\lambda +2\mu +\frac{(2l+4m+3\lambda +6\mu )\;(\lambda +\mu )\;\;}{(3\lambda \mu +{2\mu }^{2})\;}\sigma ,\;C_{12}=\lambda +\frac{(2l+\lambda )\;(\lambda +\mu )\;\;}{(3\lambda \mu +{2\mu }^{2})\;}\sigma ,\;C_{22}=\lambda +2\mu +\frac{(2l+\lambda )\;(\lambda +\mu )\;\;}{(3\lambda \mu +{2\mu }^{2})\;}\sigma ,\;C_{23}=\lambda +\frac{(2l-2m+n)\;(\lambda +\mu )\;\;}{(3\lambda \mu +{2\mu }^{2})\;}\sigma ,\;C_{55}=\mu +\frac{(m+\lambda +2\mu )\;(\lambda +\mu )\;\;}{(3\lambda \mu +{2\mu }^{2})\;}\sigma ,$$

where λ and μ are the Lamé coefficients.

**Figure 1:**
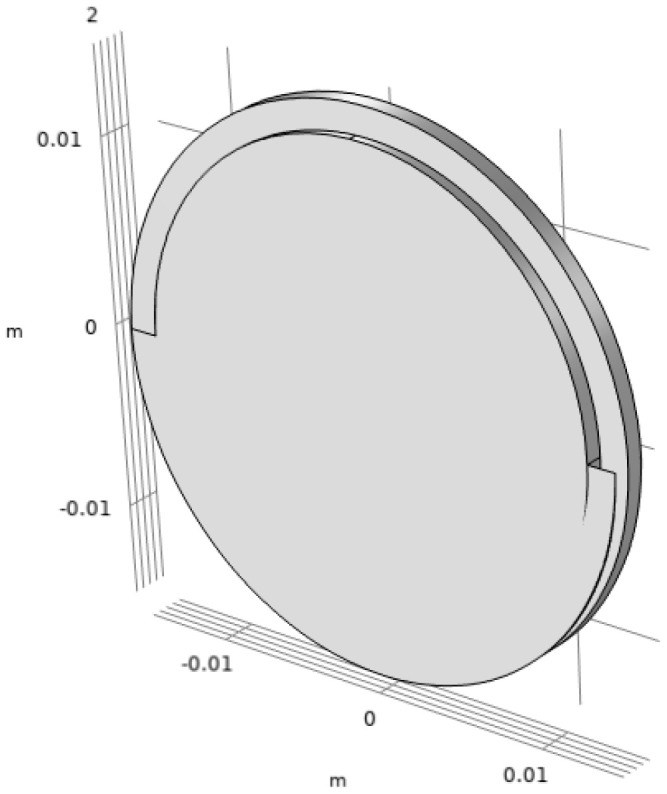
Three-dimensional geometry of the bar on a thread pitch implemented using a SAFE computation.

The model implemented in COMSOL is presented in Figure 1. There are other tools available for calculating these dispersion curves for arbitrary cross-sections [32,33], but in this study, this is a 3D model [34] representing the threaded rod with a single thread pitch to account for the effect of threading on the dispersion curves.

**Figure 2:**
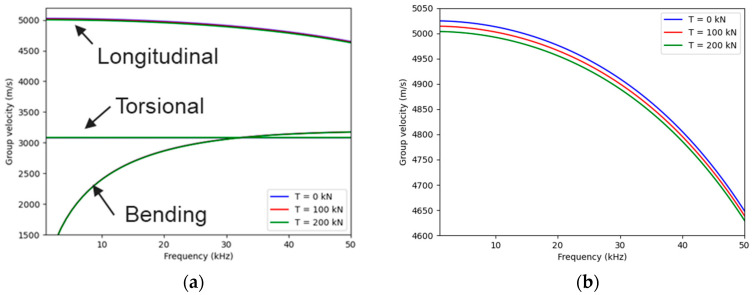
(**a**) Dispersion curves calculated using the SAFE method and (**b**) zoom on the longitudinal mode.

Dispersion curves were calculated for three different tension levels, 0 kN, 100 kN, and 200 kN, using the properties provided in Table 1, where d is the rod diameter; ρ is the density; and V_L_ and V_T_ are the longitudinal and transverse wave velocities, respectively. The results are displayed in Figure 2.

Figure 2 shows that the longitudinal mode is primarily affected by the applied tension. This result aligns with studies indicating that the sensitivity of ultrasound to tension is greater when the wave polarization is in the same direction as the loading axis [15]. The sensitivity of this mode to tension is linear and equal to −0.10 (m/s)/kN.

The measurement of this longitudinal mode would thus allow for the estimation of the effective tension in the anchor rod. However, the steel properties encountered in situ are unknown. Numerically, we can investigate the influence of these parameters. The rod diameter is fixed, and the density, longitudinal and transverse wave velocities, and steel density are studied successively.

The values of chosen parameters for the sensitivity’s determination are indicated in Table 2, and when one parameter is analyzed, the values of the other parameters are fixed and correspond to those provided in Table 1. The input parameter ranges used for wave velocities and density are primarily intended to provide an order of sensitivity estimation. They are representative of typical steel properties found in industry standards and experimental data.

**Figure 3:**
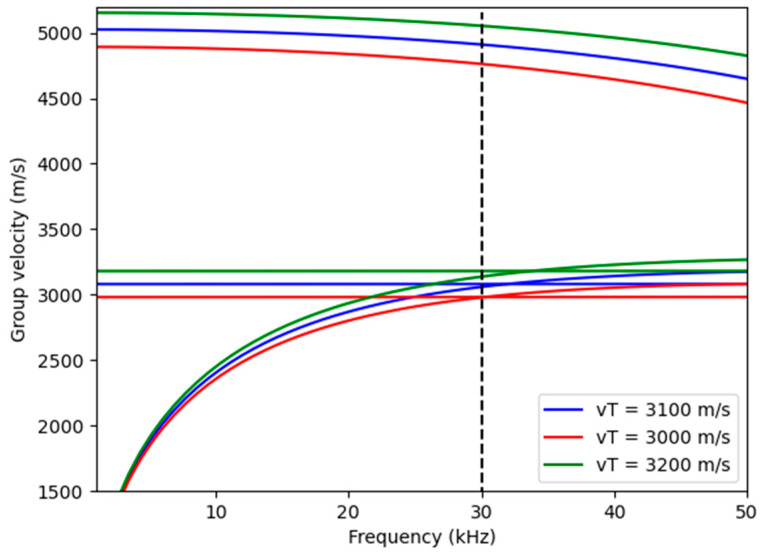
Influence of shear wave speed on longitudinal, torsional, and bending modes.

The dispersion curves for the different shear wave velocities are shown in Figure 3, while the sensitivity results at 30 kHz for the other parameters are provided in Table 3.

In Figure 3, it can be observed that all modes are particularly sensitive to the shear wave velocity.

For the longitudinal mode, the sensitivity obtained was 0.10 (m/s)/kN. Thus, a tension variation of 100 kN corresponds to a velocity variation of 10 m/s. On the other hand, a misestimation of the shear wave velocity of around 7 m/s results in the same 10 m/s variation in the longitudinal mode, which could mask the effect of tension on the longitudinal mode. Similarly, an estimation error of around 90 m/s in the longitudinal wave velocity results in the same 10 m/s variation. This highlights the critical importance of accurately estimating the shear wave velocity (on the order of 1 m/s) and, to a lesser extent, the longitudinal wave velocity (on the order of 10 m/s) to reliably estimate the tension. The effect of density is negligible, and a typical value for steel can be used.

We can also observe in this table that the sensitivities of the torsional and bending modes to tension are low. Furthermore, the torsional mode is exclusively sensitive to the shear wave velocity, while the bending mode is primarily sensitive to the shear wave velocity and slightly to the longitudinal wave velocity.

Thus, by measuring the three modes, the proposed approach in this study can use the torsional and bending modes to estimate the unknown parameters of the bar and the longitudinal mode to access information about the tension. Given the low sensitivity to stress (0.2% on the longitudinal mode), this methodology must be applied to each bar inspected in situ to account for the actual wave velocities at the time of inspection, thus minimizing the influence of external conditions.

To reduce measurement uncertainty, the idea is to reconstruct these dispersion curves over a wide frequency range.

### 2.2. Experimental Setup

**Figure 4:**
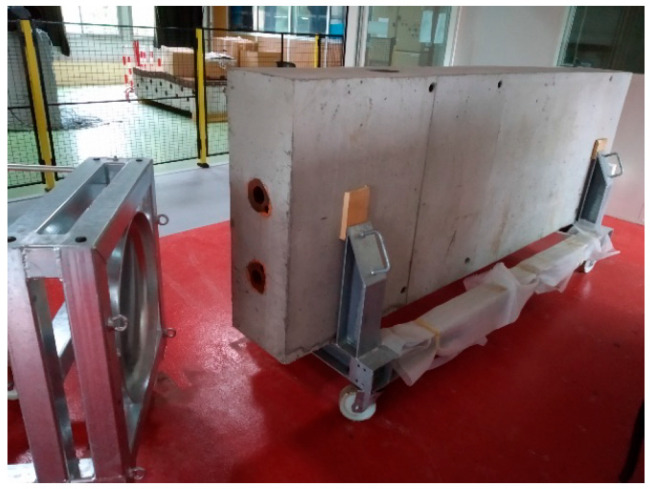
Concrete beam measuring 3 m × 1 m × 0.4 m with two slots for the insertion of tie rods.

**Figure 5:**
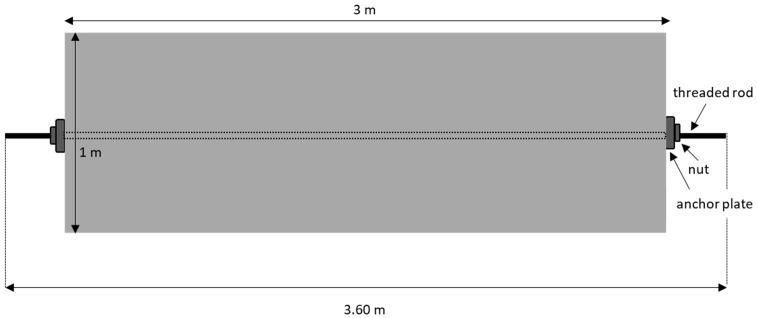
Diagram of the test setup for tensioning the bar.

To carry out this study under controlled conditions, a 3 m long concrete beam equipped with two ducts was fabricated (Figure 4). In one of these ducts, a 3.60 m long threaded steel bar with a diameter of 28.6 mm was placed (Figure 5).

The measurements are performed in reflection mode, with the transmitter and receiver placed on the same side at the end of the bar.

The emission and reception configurations are chosen to preferentially measure the desired mode to be generated and analyzed. For all three measurements, excitation is applied to the cross-section of the bar, and the receiving sensor is placed on the first thread pitch at the end of the bar.

All the sensor configurations are summarized in Table 4. The references for the P-wave and S-wave sensors are ACS S1905 and ACS S1802 (Acoustic Control Systems—ACS Group, Saarbrücken, Germany), respectively. These are dry-point contact sensors that do not require gel-type coupling or any special surface treatment. S-wave transducers are employed for measurements because they can preferentially detect the longitudinal or transverse components of displacements based on their orientation [1]. The longitudinal mode exhibits only a longitudinal component, the torsion mode has a solely transverse component, and the bending mode includes components in both directions. Therefore, mode selection is achieved by judiciously orienting the S-wave transducer. In the configuration used to measure the longitudinal mode, the transmitter sensor is a P-wave sensor, and the receiver sensor is an S-wave sensor polarized along the axis of the bar to preferentially capture the displacement component corresponding to the longitudinal mode.

To generate the torsional mode, two transmitter sensors are used, both positioned on the cross-section of the bar and emitting in phase opposition to apply a local torsional movement and enhance torsional motion. The receiver sensor is an S-wave sensor oriented 90° relative to the axis of the bar to measure the transverse displacement component of this mode.

Finally, to preferentially measure the bending mode, an S-wave sensor is used during emission, while another S-wave sensor oriented at 45° relative to the axis of the bar is used during reception to measure displacement components in both directions.

The transmitted signal is a frequency-modulated signal ranging between 10 kHz and 35 kHz with a duration of 10 ms. The frequency band was chosen based on the passbands of the sensors used. The signal is generated by a TiePie HS5 function generator (TiePie Engineering, Sneek, The Netherlands) with a maximum voltage of 12 V. A fixed-gain amplifier of 20 dB is used for transmission, and the input voltage is adjusted to optimize the signal-to-noise ratio.

To reconstruct the dispersion curves associated with each mode, for each measurement, the cross-correlation between the transmitted and received signals is calculated, and a time–frequency analysis using a spectrogram is applied.

The spectrogram was calculated using a Hann window, a segment duration of 700 µs, and maximum overlap, with only a one-sample gap between successive segments.

The accuracy of the obtained dispersion curves could have been influenced by signal reflections from other components, such as the anchor plate, because the extraction of dispersion curves, as described in the next section, relies on selecting echoes corresponding to the wave propagation of a unique mode over twice the length of the bar. Additionally, measuring multiple echoes with a good signal-to-noise ratio enhances the accuracy of dispersion curves.

## 3. Results

**Figure 6:**
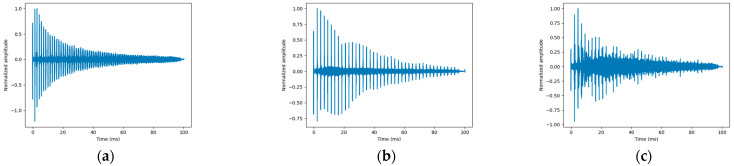
Intercorrelation results for each configuration: (**a**) compression, (**b**) torsional, and (**c**) bending.

The cross-correlation results associated with each measurement are shown in Figure 6.

Each measurement was performed to maximize the excitation and measurement of a single mode. In these correlation signals, various echoes related to the propagation time of the wave over twice the length of the bar are visible. In Figure 6a, the echoes are closer together compared to the measurement results in Figure 6b,c, where torsional and bending modes are preferentially expected given the higher velocity of the longitudinal mode. In Figure 6c, as the wave propagates back and forth, the echoes become less distinct than the other modes. This is explained by the fact that the bending mode is highly dispersive, resulting in a wave packet that spreads out over time. We should recall that the transmitted signal is a frequency-modulated signal ranging from 10 kHz to 35 kHz with a duration of 10 ms. The dispersion of the bending mode is particularly significant over this frequency range. Additionally, mode conversions can occur with each reflection, adding extra signals between each echo. Overall, the signal-to-noise ratio remains satisfactory.

Based on the first three correlation maxima, the average propagation time over this distance is estimated for each measurement. The results are indicated in Table 5. The results in the table are consistent with theoretical expectations based on the dispersion curves. The propagation time, ΔT, is shortest for the longitudinal mode (1.4 ms), followed by the torsional mode (2.4 ms) and the bending mode (2.4 ms). For the bending mode, the first arrival time is close to that of the torsion mode because, in a frequency range of around 30 kHz, their dispersion curves intersect. Below this range, the velocity is lower for the bending mode, which explains why the wave packet is more spread out.

With this type of representation, the spectral content is not accessible. Therefore, to analyze the dispersion of each mode, the spectrogram is calculated for each signal, and the duration intervals (ΔT) are used to select each echo. The first 10 echoes are selected for each measurement.

**Figure 7:**
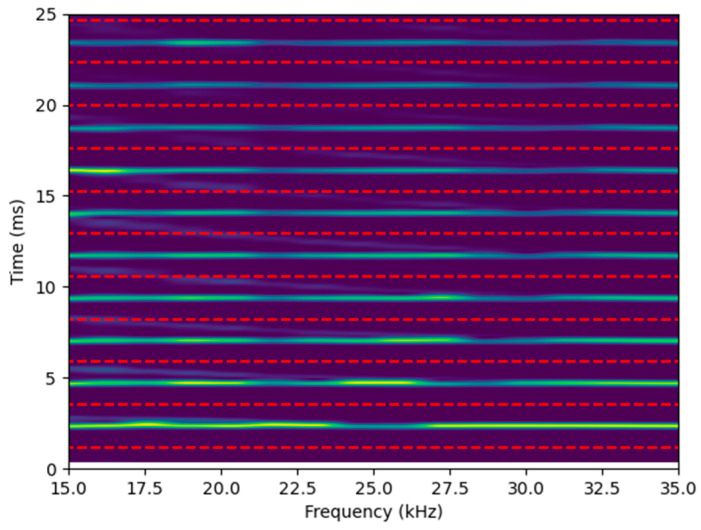
Spectrogram obtained for the configuration that preferentially measures the torsional mode, with red dashed lines indicating the limits for selecting each echo.

For example, in Figure 7, corresponding to the spectrogram of the measurement designed to preferentially capture the torsional mode, the red dashed lines represent the lower and upper boundaries used to select each wave reflection. The first dashed line is positioned at the first correlation peak minus ΔT/2, and each subsequent red dashed line is spaced by ΔT, marking successive wave reflections.

Within each band defined by two red lines, the maximum amplitude at each frequency is extracted. Although multiple modes may be present within the selected band, the torsional mode has been predominantly excited and measured. Consequently, other modes are disregarded, ensuring the isolation of the mode of interest.

**Figure 8:**
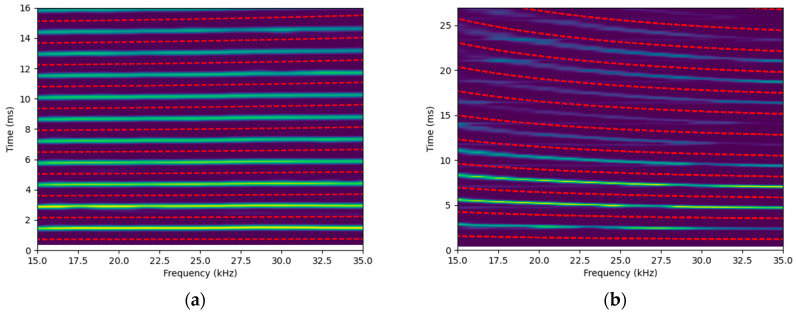
Spectrograms obtained for the configuration that preferentially measures the (**a**) longitudinal mode and (**b**) bending mode, with red dashed lines indicating the limits for selecting each echo.

Since the torsional mode is nearly non-dispersive, the propagation cycles can be delineated using evenly spaced lines at intervals of ΔT. However, the longitudinal and bending modes are much more dispersive, meaning that propagation cycles cannot be selected using simple straight lines. Instead, theoretical dispersion curves for standard steel are used to determine the expected shape of the longitudinal and bending modes in the time domain, based on the fixed bar length. The same procedure is then applied by shifting these curves by ΔT and extracting the maxima for each frequency. Note that the strong dispersive nature of the bending mode (Figure 8b) becomes increasingly apparent in the time–frequency domain, as the time difference due to dispersion accumulates with distance. If straight lines had been used to select the echoes, the selection of the signal associated with the bending mode would have been affected.

Finally, the dispersion curves can be reconstructed by converting the frequency–time representation into a frequency–group velocity ($v_{g}$) representation. This is achieved by considering, for each band, the distance traveled, as defined by the equation below:

(3) $$v_{g}(f)=\frac{2nL}{t_{n}(f)}$$

where L is the length of the bar, f is the frequency, and n represents the number of propagation cycles ranging from 1 to N=10.

**Figure 9:**
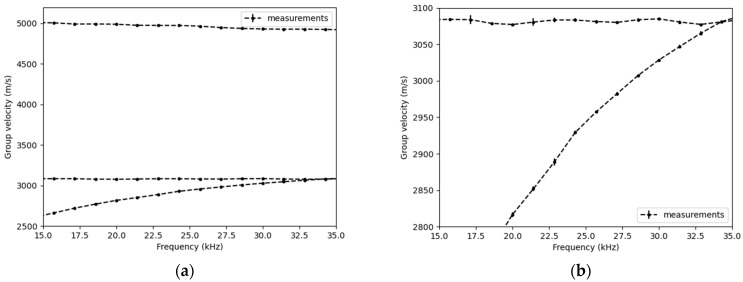
Experimental dispersion curves with (**a**) between 15 kHz and 35 kHz and (**b**) a zoom on the torsional and bending modes.

Thus, for each mode and each frequency, there are 10 measurements. The result is represented by averaging the N=10 points for each frequency, with an error bar corresponding to the mean standard deviation. The result is displayed in Figure 9.

The applied methodology enabled the reconstruction of the three dispersion curves with unilateral access at the end of the rod, even for the highly dispersive bending mode. Since the error bars are small compared to the scale of Figure 9b, a zoom on the bending and torsional modes allows for their visualization. Table 6 shows the minimum, maximum, and average values of standard deviation for the different dispersion curves between 15 kHz and 35 kHz.

The minimum error value obtained is on the order of 1 m/s, which is satisfactory relative to the target uncertainties. For certain frequencies, this error is more significant, reaching a maximum value of 14.4 m/s for the longitudinal mode, 5.8 m/s for the torsional mode, and 9.4 m/s for the bending mode. On average, these errors remain relatively low, with the average error for the torsional mode being 2.5 m/s, allowing for the precise determination of the transverse wave velocity. Overall, the results obtained, which can be further optimized, provide a solid foundation for estimating the effective tension in the bar.

**Figure 10:**
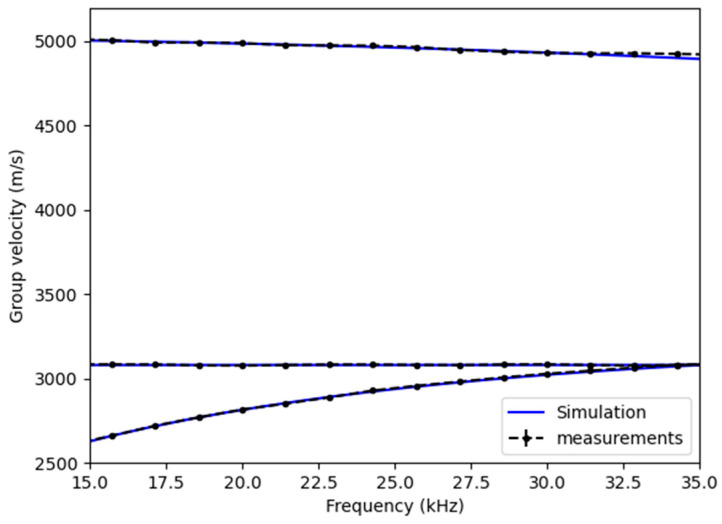
Comparison of simulated and experimental curves.

Now, we propose fitting the experimentally obtained dispersion curves as closely as possible with the simulation results. The parameters used for the simulation are listed in Table 7, and a comparison is shown in Figure 10.

Figure 10 shows that there is very good consistency between the experimental and simulation results. The average deviation between the measured and simulated curves over the entire frequency range is shown for each mode in Table 8.

The deviations are relatively small and can be explained by measurement uncertainties, particularly related to the rod’s diameter, its length, and the experimental setup in general. Moreover, in the simulation, the model considers only a threaded rod, while the nuts and anchor plates influence the propagation of guided waves.

Experimental accuracy could be improved by using sensors with varying bandwidths to reconstruct the curves over a wider frequency range, thereby reducing uncertainties. The measurement of additional higher-order modes could also be a possibility to explore, with the goal of increasing redundancy and accurately determining all unknown parameters.

In terms of data processing, analysis tools such as the Wavelet Transform [35] or the Wigner–Ville distribution [36] could provide a better trade-off between time and frequency resolution.

## 4. Conclusions

In this study, a non-destructive method based on the use of guided ultrasonic waves was investigated to estimate the effective tension of anchor rods.

The simulation results identified the longitudinal mode as sensitive to tension and quantified this sensitivity. A numerical parametric study demonstrated the importance of accurately knowing the stress-free velocity properties of steel to enable the reliable tension estimation of the rod.

This study, conducted under controlled conditions, enabled the implementation of a methodology for extracting three dispersion curves corresponding to the longitudinal, torsional, and bending modes. These curves were constructed over a frequency range of 15 kHz to 35 kHz.

This methodology, applicable through measurements at the rod’s end, would enable the determination of the rod’s properties, which could then be used to estimate the effective tension without a previous calibration step.

Ongoing work aims to estimate the stress-free properties of steel by analyzing the first few centimeters of rods accessible before the anchor plate. High-frequency measurements at 10 MHz using bulk waves could help determine longitudinal wave velocity in the stress-free state. Thus, knowing this velocity in the free section would eliminate one unknown, and combining information from different modes would enable the determination of the rod’s length, transverse wave velocity, and tension.

It is worth noting that the dispersion curves are also sensitive to the rod’s diameter, which results in a shift in the curves along the frequency axis. This parameter could also be treated as an unknown, providing insights into the corrosion state of passive anchor rods, as general corrosion typically causes an overall reduction in diameter. An optimization algorithm could be implemented to adjust the various unknowns.

An uncertainty analysis is also planned to assess whether the proposed methodology, based on multiple indirect measurements, is compatible with the accuracy required for absolute effective tension estimation.

Challenges also arise when applying this to real structures with potentially rusted bars. The attenuation could be more significant than in laboratory conditions. Ongoing work aims to achieve better representativeness and evaluate the robustness of the proposed method.

## Figures and Tables

**Table 1:** Properties of the threaded steel bar.

d (mm)	Thread Height (mm)	ρ (kg/m^3^)	V_L_ (m/s)	V_T_ (m/s)	Murnaghan Coefficients (l, m, n) × 10^3^ MPa
27	1.6	7950	5950	3100	(−932; −335; −1025)

**Table 2:** Values of the input parameters for sensitivity estimation.

Parameter	ρ (kg/m^3^)	V_L_ (m/s)	V_T_ (m/s)
Initial value	7950	5950	3100
Lower limit	7850	5850	3000
Upper limit	8050	6050	3200

**Table 3:** Sensitivity results for tension (S_T_), density (S_ρ_), longitudinal velocities (S_vL_), and shear wave velocities (S_vT_).

Parameter	S_T_ (m/s)/(kN)	S_ρ_ (m/s)/(kg/m^3^)	S_vL_ (m/s)/(m/s)	S_vT_ (m/s)/(m/s)
Longitudinal mode	−0.10	~10^−11^	0.11	1.45
Torsional mode	0.02	~10^−11^	1.3 × 10^−4^	0.99
Bending mode	−0.02	~10^−11^	0.04	0.79

**Table 4:** Configurations to preferentially excite one mode.

Configuration	Longitudinal	Torsional	Bending
Transmitter	P-Wave	2 S-Wave|90°/−90°	S-Wave|90°
Receiver	S-Wave|0°	S-Wave|90°	S-Wave|45°

**Table 5:** Average propagation time over distance.

Expected Mode	∆T (ms)
Longitudinal mode	1.4
Torsional mode	2.4
Bending mode	2.4

**Table 6:** Minimum, maximum, and average values of standard deviations.

Parameter	Min (std/$\sqrt{N}$) (m/s)	Max (std/$\sqrt{N}$) (m/s)	Mean (std/$\sqrt{N}$) (m/s)
Longitudinal mode	1.3	14.4	7.6
Torsional mode	1.0	5.8	2.5
Bending mode	1.5	9.4	4.3

**Table 7:** Model parameters used to fit the simulated and measured curves.

d (mm)	Thread Height (mm)	ρ (kg/m^3^)	V_L_ (m/s)	V_T_ (m/s)
27	1.6	7950	5965	3100

**Table 8:** Mean deviation of each mode between 15 kHz and 35 kHz.

Parameter	Mean Deviation (m/s)
Longitudinal mode	6.3
Torsional mode	2.2
Bending mode	4.3

## Data Availability

The data presented in this study are available upon request from the corresponding author due to privacy reasons.
